# Supplementary material for: Thermoelasticity of ice explains widespread damage in dripstone caves during glacial periods
Source: Sci Rep. 2023 May 6;13:7407. doi: 10.1038/s41598-023-34499-9 (PMC10164159; doi:10.1038/s41598-023-34499-9)
Supplement: Supplementary file 1 — Supplementary Information. [file 41598_2023_34499_MOESM1_ESM.pdf]

10.1038/s41598-023-34499-9

File Correction Details

Correction is made. No of Corrections: 20

Online Correction Link: **https://eproofing.springer.com/ePj/index/n0D5jQ2Hki-6vU4wOJhffLCKSx66JnIo7yT5F5WDpkEFtYL7eVDhAgsS-0OSn-vEQwyIT3R5JyBAiyHbrQ0tuZzHp5SEZt2CNhQUczsAZSMHSJwrsyMfuWj2ADmUkhh_**

Image Annotation Details

No Details Found

Attached File Details

No Details Found

Query Details

1. **Please note that tables were not sequentially cited in the text, and have been renumbered in the text. Please check, and correct if necessary.**
   checked and corrected a few mistakes

2. **Please confirm the section headings are correctly identified.**
   ok

Article

Thermoelasticity of ice explains widespread damage in dripstone caves during glacial periods

Open Access This article is licensed under a Creative Commons Attribution 4.0 International License, which permits use, sharing, adaptation, distribution and reproduction in any medium or format, as long as you give appropriate credit to the original author(s) and the source, provide a link to the Creative Commons licence, and indicate if changes were made. The images or other third party material in this article are included in the article's Creative Commons licence, unless indicated otherwise in a credit line to the material. If material is not included in the article's Creative Commons licence and your intended use is not permitted by statutory regulation or exceeds the permitted use, you will need to obtain permission directly from the copyright holder. To view a copy of this licence, visit http://creativecommons.org/licenses/by/4.0/.

Christoph Spötl ✉ Email : christoph.spoetl@uibk.ac.at Affiliationids : Aff1, Correspondingaffiliationid : Aff1

Alexander H. Jarosch Affiliationids : Aff2

Andreas Saxer Affiliationids : Aff3

Gabriella Koltai Affiliationids : Aff1

Haiwei Zhang Affiliationids : Aff4

Aff1 Institute of Geology, University of Innsbruck, 6020, Innsbruck, Austria

Aff2 ThetaFrame Solutions, Hörfarterstrasse 14, 6330, Kufstein, Austria

Aff3 Department of Structural Engineering and Material Sciences, University of Innsbruck, 6020, Innsbruck, Austria

Aff4 Institute of Global Environmental ChangeXi'an Jiaotong UniversityXi'an, 710054, China looks like there is an unnecessary blank after "Xi'an" and before the zip code; please check

Received: 4 February 2023 / Accepted: 3 May 2023

Abstract

Damage to speleothems is a common phenomenon in mid-latitude caves, and multiple causes have been proposed. Here we report on one of such type of damage, namely stalagmites that are broken and partially sheared near their base but are still in upright position. Such stalagmites occur in the Obir Caves (Austria) associated with cryogenic cave carbonates, demonstrating the former presence of cave ice. ^{230}Th dating suggests damage to the speleothems during the Last Glacial Maximum. Numerical modelling combined with laboratory measurements demonstrates that internal deformation within a cave ice body cannot fracture stalagmites, even on a steep slope. Instead, temperature changes lead to thermoelastic stresses within an ice body that reach values equaling to and exceeding the tensile strength of even large stalagmites. Differences in thermal expansion coefficients cause a sharp vertical jump in stress between the stalagmite and the surrounding ice body, and the ice lifts the stalagmite as it expands with increasing temperature. This study refutes the previously accepted model that flow of ice breaks stalagmites, and suggests a link between glacial climate variability and corresponding cooling and warming cycles in the subsurface that weaken and eventually fracture stalagmites due to the opposing thermoelastic properties of calcite and ice.

SECTION 1

Introduction

Speleothems are secondary mineral deposits in caves, usually composed of calcium carbonate. They come in a variety of shapes and dimensions, with stalactites (growing from the ceiling), stalagmites (growing upward from the floor), and flowstones (sheet-like formations) being the most common types. In addition to their aesthetic aspect (e.g. in show caves), speleothems are also of high scientific value as archives of past environmental and climate changes[1]. Radiometric techniques, particularly ^{230}Th dating[2], allow speleothems to be used as accurate chronometers for the past of approximately 600 ka (ka represents thousands of years before present, with the present referring to 1950 AD).

In addition to paleoclimate research, speleothems from caves in seismically active regions have also been used as recorders of strong earthquakes in the past. In several studies, intensities and ground accelerations of paleoseismic events were derived from damaged stalagmites, partly calibrated by measurements of petrophysical properties of speleothems[3, 4, 5, 6, 7, 8]. Dating the youngest calcite of a seismically fractured speleothem provides a maximum age constraint for the seismic event. Combining this with a date for calcite healing these fractures and/or growing on top of such a fallen formation offers a way to bracket the age of the suggested earthquake in the past[7, 9].

Damage of speleothems, however, can have other causes as well, such as severe cave flooding, mechanical instabilities (e.g. ceiling collapse, overturning of stalagmites due to erosion of underlying clastic sediments) or destruction by humans[10, 11].

A final process that can cause damage to speleothems is deformation by ice that has accumulated in caves during cold climate periods. Although not previously reported from modern ice-bearing caves, there are a number of observations in studies from currently ice-free caves that strongly suggest that ice damage to speleothems is a widespread phenomenon in mid-latitude caves. These include (a) stalagmites sheared from the base, some still upright, some titled or overturned, or lying on the floor, (b) stalactites broken at some level across a cave chamber, (c) precariously placed fragments of speleothems or bedrock attached (cemented) to steep cave walls, (d) cracked conical stalagmites, (e) fractured flowstones, and (f) shattered stalagmites[12, 13, 14, 15, 16]. These observations were primarily made in mid-European and some British caves where other processes such as strong earthquakes were regarded as unlikely given their stable tectonic setting. In several of these caves, there is independent evidence for the presence of ice deposits provided by the close association of damaged speleothems and accumulations of coarse grained cryogenic cave carbonates (CCC[17]). The stable isotopic composition of these CCC indicates crystallization in very slowly freezing pools of water in cave ice[18].

While these empirical observations are important and generally consistent with independent paleoclimatic interpretations suggesting wide-spread permafrost in regions such as Central Europe during very cold climate epochs of the last glacial period[19, 20, 21], systematic studies investigating the deformation of speleothems by ice are lacking. Furthermore, to our knowledge, no attempt has been made to numerically model this type of mechanical deformation, with the exception of a short report suggesting that thermal expansion of ice due to warming could exert sufficient mechanical force to shear stalagmites from their base[14].

The aim of this study was to combine field observations from a cave showing damaged speleothems with numerical simulations calibrated with rock-mechanical parameters obtained from speleothems of the same cave to investigate the process of speleothem deformation by ice. We selected a cave system where deformed speleothems are spatially associated with CCCs formed during the Last Glacial Maximum, and focused on stalagmites because of their well-defined geometry.

SECTION2

Study site

The Obir Caves comprise a set of caves in the Northern Karawank Mountains of southern Austria, near the border with Slovenia (Suppl. Fig. 1). These caves are hydrologically inactive and were formed in Middle to Upper Triassic limestone by upwelling groundwater enriched in carbon dioxide followed by tectonic uplift[22]. Because of their hypogene origin, these caves had no natural entrances and where only discovered during ore mining in the nineteenth century. Modern air temperature in the Obir Caves is close to 6 °C, which corresponds to the mean annual air temperature outside at the elevation of the caves (approximately 1000–1100 m a.s.l.).

Many parts of Obir Caves are decorated with stalactites, flowstones and stalagmites and parts of these caves are a show cave. ^{230}Th dating of stalagmites show that they formed during interglacials and major interstadials of the past 600 ka (Ref.[23]and unpublished data by the authors).

In parts of these labyrinthic caves, CCC are present that formed between 26.6 and 23.5 ka BP[24]. Thus, during this interval, which corresponds to the first part of the Last Glacial Maximum, perennial ice deposits occupied parts of these caves that where close to the 0 °C isotherm and allowed precipitation of CCC. In this study, we focus on the Banane system of Obir Caves, a fairly pristine part of these caves where CCC are spatially associated with deformed stalagmites. The caves of the Banane system are between about 49 and 67 m below the surface.

Other parts of Obir Caves have recently been studied in the context of paleoseismicity because the cave is located near the Periadriatic Lineament, a major seismically active strike-slip fault. These studies provided evidence for speleothem fracturing associated with movement along faults[8], providing a unique opportunity of compare these deformation features with those associated with the past presence of ice.

SECTION1

Results and discussion

SECTION2

Field observations

Stalagmites are found in several places in the Banane system and are mostly slightly conical in shape and less than half a meter high. Slim equal-diameter (candle-stick type) stalagmites with height/width ratios > 10 are not present in Banane system and are also rare in other parts of the Obir Caves. The pre-Holocene speleothem generations in the Obir Caves are relatively easy to distinguish from the Holocene ones by their color (yellowish vs. white). Deformation features were observed only in Pleistocene stalagmites, i.e. yellowish stalagmites, that are inactive and locally overgrown by young whitish calcite (Fig. 1). Mapping of the Banane system has shown that these fractured stalagmites occur in chambers and galleries near CCC sites dated to the Last Glacial Maximum (Suppl. Fig. 1). In some of these places CCC are already cemented by a thin layer of calcite, similar to some fractured stalagmites, whose cracks are partially filled or covered by younger calcite. In the parts of the Banane system where fractured stalagmites are present, there is no evidence of exposed fault planes, fresh slickensides, or any other indications of neotectonic movement in the limestone bedrock.

Figure 1

Examples of stalagmites from the inclined chamber south of Sandgang (see Suppl. Fig. 1) showing evidence of mechanical breakage and results of ^{230}Th dating. Red arrows mark fracture planes partly healed by later calcite. Yellow dots show locations of drill cores with ages of pre- and post-deformation calcite in ka BP. (**A**) Stalagmite twin with basal fracture which offsets the formation by up to 2 cm in downslope direction. Black arrows mark precariously placed fragments broken off the base of the larger stalagmite (samples Obi-B5). The oblique drill core penetrated a thin layer of calcite that grew across the fracture. (**B**) Stalagmite growing on a steep flowstone substrate and cut by two subhorizontal fractures. Note younger calcite on the left side concealing the fractures (samples left: Obi-B1; samples right: Obi-B2). The upper drill core first penetrated a thin layer of this younger calcite, while the lower drill core encountered a thicker layer of this calcite whose basal age is early Holocene, underlain by pre-fracture calcite. (**C**) Stalagmite completely broken off its base and cemented by younger calcite. The post-fracture stalagmite growing on top of the fallen stalagmite was drilled and returned at Late Glacial age at its base (samples Obi-B3).

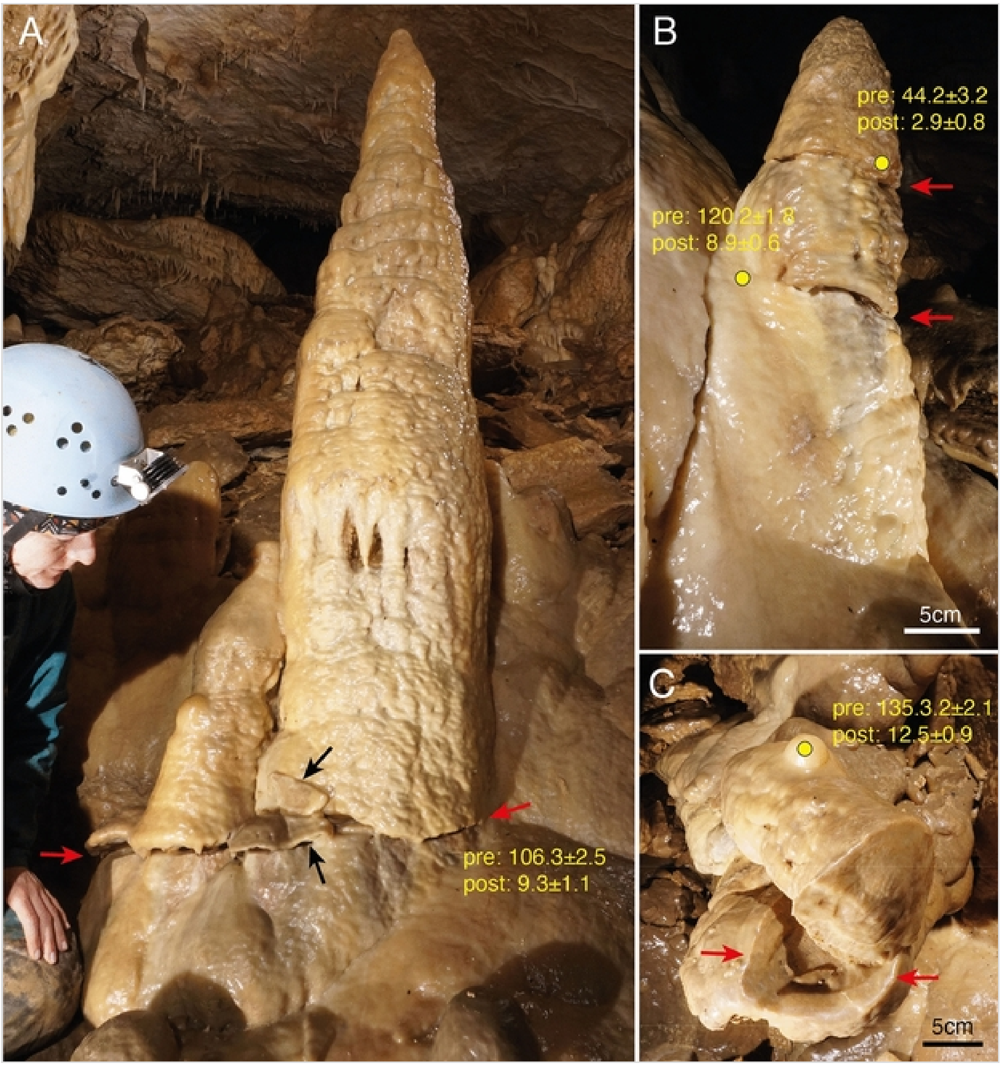

Most stalagmites studied show a subhorizontal crack near their base, but some show such a crack higher up (near half height). Few examples of oblique fractures were found, probably due to the angle of the inclined substrate. One stalagmite shows two fractures at different heights (Fig. 1B). Roughly half of the examined stalagmites show no lateral displacement of the upper part with respect to the lower part, while the other half show clear signs of displacement along the fracture. The largest offset was measured at the base of the largest stalagmite (about 2 cm – Fig. 1B). The azimuth of the displacement always corresponds to the direction of inclination of the sloping substrate on which the speleothem grew. In about half of the damaged in-situ stalagmites, at least some younger calcite is present to hold the broken pieces together.

Besides fractured stalagmites, we also observed stalagmites and fragments of them scattered on the ground. Broken stalactites are even more common in some parts of the Banana system, while fractured flowstones have been observed in only a few places. These damaged speleothems have not been studied in detail, as their origin can be less reliably associated with the past presence of ice. In a few places, fragments of speleothems attached (cemented) precariously to fractured stalagmites are also present (Fig. 1 A).

Age constraints

Three stalagmites showing post-fracture calcite were sampled using small-diameter drilling; results are shown in Fig. 1 and listed in Table 1. The post-fracture calcite yielded ages between the Late Glacial and the Late Holocene, while pre-fracture calcite has a much older age between 135 and 44 ka. Due to the presence of impurities and low U content, the precision of the post-fracture calcite ages is comparably low.

Table 1 ^{230}Th dating results.

Sample	^{238}U	^{232}Th	^{230}Th/^{232}Th	δ^{234}U*	^{230}Th/^{238}U	^{230}Th Age (yr)	^{230}Th Age (yr)	$\delta^{234}U_{Initial}$**	^{230}Th Age (yr BP)***
Number	(ppb)	(ppt)	(Atomic x 10^{-6})	(Measured)	(Activity)	(Uncorrected)	(Corrected)	(Corrected)	(Corrected)
Obi-B1a	109.9 ± 0.2	3610 ± 73	50 ± 1	162.1 ± 3.5	0.1002 ± 0.0021	9816 ± 216	8993 ± 620	166 ± 4	8921 ± 620
Obi-B1b	96.9 ± 0.2	6505 ± 131	203 ± 4	198.3 ± 3.0	0.8269 ± 0.0051	121769 ± 1453	120231 ± 1798	278 ± 4	120159 ± 1798
Obi-B2a	101.0 ± 0.2	4471 ± 90	16 ± 1	185.9 ± 2.9	0.0428 ± 0.0018	4007 ± 176	2918 ± 790	187 ± 3	2846 ± 790
Obi-B2b	68.6 ± 0.1	12588 ± 253	39 ± 1	197.6 ± 3.5	0.4365 ± 0.0045	48753 ± 647	44302 ± 3214	224 ± 5	44230 ± 3214
Obi-B3a	85.9 ± 0.3	4163 ± 85	46 ± 1	144.1 ± 10.3	0.1366 ± 0.0029	13833 ± 340	12599 ± 934	149 ± 11	12527 ± 934
Obi-B3b	92.8 ± 0.2	901 ± 19	1379 ± 30	119.7 ± 3.7	0.8115 ± 0.0056	135611 ± 2061	135371 ± 2063	175 ± 6	135299 ± 2063
Obi-B5a-2	132.9 ± 0.2	8334 ± 167	30 ± 1	178.5 ± 3.1	0.1125 ± 0.0026	10918 ± 266	9366 ± 1130	183 ± 3	9294 ± 1130
Obi-B5c	59.3 ± 0.1	7191 ± 145	110 ± 2	242.1 ± 5.0	0.8068 ± 0.0065	109039 ± 1655	106350 ± 2494	327 ± 7	106278 ± 2494

The error is 2 sigma.

U decay constants: $\lambda_{238} = 1.55125 \times 10^{-10}$ (Jaffey et al. [42]) and $\lambda_{234} = 2.82206 \times 10^{-6}$ (Cheng et al. [2]). Th decay constant: $\lambda_{230} = 9.1705 \times 10^{-6}$ (Cheng et al.[2]).

Corrected ^{230}Th ages assume the initial ^{230}Th/^{232}Th atomic ratio of 4.4 ± 2.2 x 10^{-6}. Those are the values for a material at secular equilibrium, with the bulk earth ~~232~~^{232}Th/~~238~~^{238}U value of 3.8.

The errors are arbitrarily assumed to be 50%.

*$\delta^{234}U = ([^{234}U/^{238}U]_{activity} - 1) \times 1000$. **$\delta^{234}U_{initial}$ was calculated based on ^{230}Th age (T), i.e. $\delta^{234}U_{initial} = \delta^{234}U_{measured} \times e^{\lambda_{234} \times T}$.

***BP stands for Before Present, i.e. before the year 1950 AD.

SECTION2

Mechanical properties

The broken stalagmites collected for laboratory testing are composed of dense calcite with a compact, columnar fabric. This is consistent with stalagmite samples obtained from other parts of the Obir Caves that show the same type of calcite crystal fabric regardless of age[23, 25, 26]. The same type of calcite was also observed in the small-diameter drill cores obtained from damaged stalagmites in Banane cave.

The tensile and compressive strengths exhibit high standard deviations of 35% and 54%, respectively, indicating a large variability in the strength properties of the measured stalagmite samples. The differences are most likely due to inhomogeneities of the stalagmites reflecting the uneven growth structure and/or the possible presence of microcracks as these specimens were detached from their substrate (unknown fracture process). Varying densities and associated porosities, as shown by the measured density variation of the samples, significantly affect strength performance, especially when small sample geometries are examined.

The Young's modulus in tension is about 9% lower than in compression (Table 2). The standard deviation for tension is 11% and for compression 14% which is much lower compared to the strength values. Similar standard deviations were found for the Poisson ratios with 15% in tension and 7% in compression.

Table 2 Mechanical properties of stalagmite samples given as mean values.

	Tension			Compression			Density
	Tensile strength	Young's modulus[3]	Poisson ratio[3]	Compressive strength	Young's modulus[4]	Poisson ratio[4]	
	MPa	GPa		MPa	GPa		kg/dm^3
Mean	4.3	64.1	0.27	56.1	72.9	0.29	2.67
std[1]	1.5	7.3	0.04	30.2	10.3	0.02	0.02
Minimum	1.0	53.7	0.21	16.5	53.2	0.27	2.63
Maximum	6.1	80.9	0.35	99.1	88.8	0.35	2.68
n[2]	16	19	16	8	23	10	10

[1]Standard deviation.

[2]Number of measurements.

[3]Stress range 0.17–3 MPa for Young's modulus and Poisson ratio.

[4]Stress range 0.50–17 MPa for Young's modulus and Poisson ratio.

The mean value of the tensile strength of the investigated stalagmite material is 4.3 ± 1.5 MPa. This value compares well with literature values for speleothems and limestone (Fig. 2).

Figure 2

Measured tensile strength of stalagmite calcite from Obir Caves (mean ± 1 standard deviation) compared to published ranges of data of stalagmites from caves in Belgium[36], Hungary, Slovakia and Bulgaria[49], stalactites from British[50] and Swiss caves[51], as well as limestone samples (compiled by[52]).

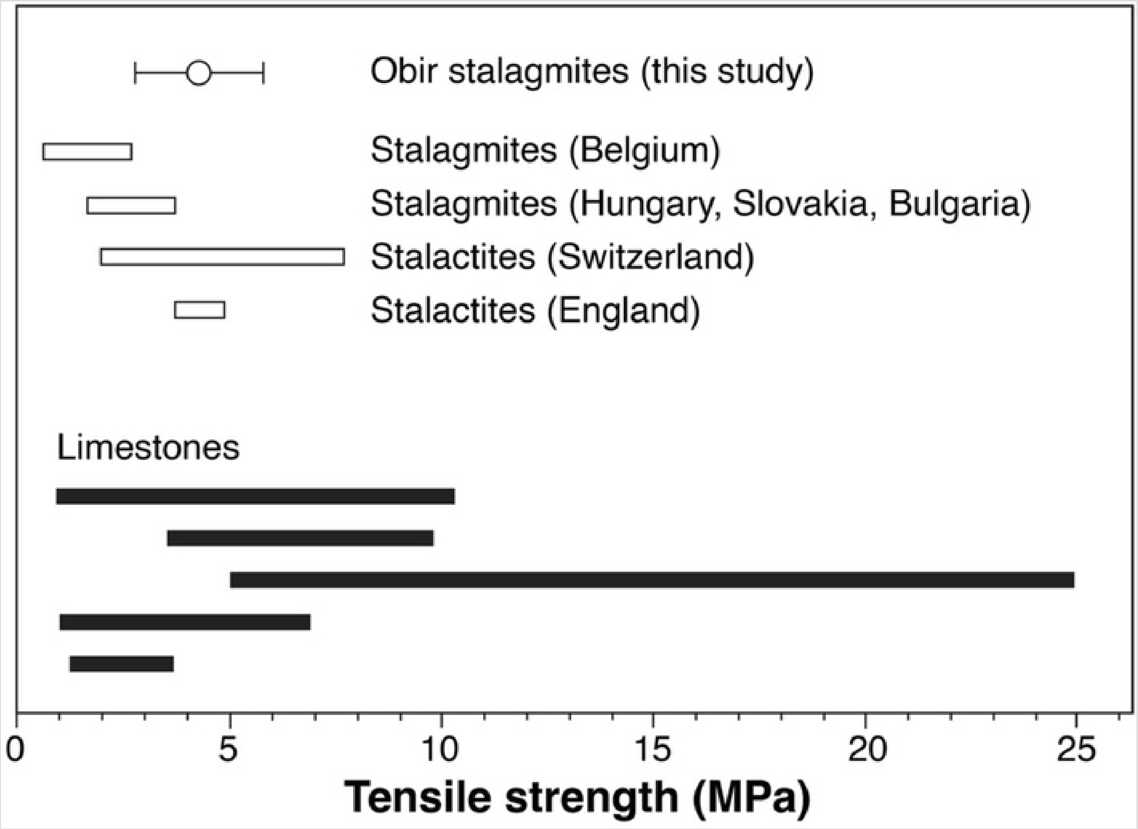

Although the results of the stalagmite parts studied show considerable variation in mechanical properties, it can be assumed that the entire stalagmites have mechanical properties that correspond to the mean values determined by these measurements.

SECTION2

Modelling results

SECTION3

Ice deformation under gravity

First, we explored the potential of ice flow to shear off stalagmites. To do this, we created a rectangular geometry with the coordinate system centered on the stalagmite (Fig. 3). Ice thickness h_{ice} and slope angle β_{ice} were varied, while the stalagmite diameter d_s was fixed at 0.4 m, as were the overall X and Y dimensions ($-7 \le X \le 14$ m and $-3.5 \le Y \le 3.5$ m). No-slip boundary conditions we applied to the side walls of the domain (i.e. the hypothetical cave walls) and to the stalagmite. At the lower boundary, we allowed free sliding along the bed to simulate the end-member case of a fully lubricated bed, which will result in the highest stresses at the stalagmite. At the upper boundary, we defined a stress-free surface boundary to allow free movement of the ice surface.

Figure 3

Schematic ice flow geometry and coordinate system of the numerical model. The light gray box shows the ice body in which the stalagmite (illustrated by the hole) is centered. This example shows the geometry for $h_{ice} = 1.0$ m and $\beta_{ice} = 10°$.

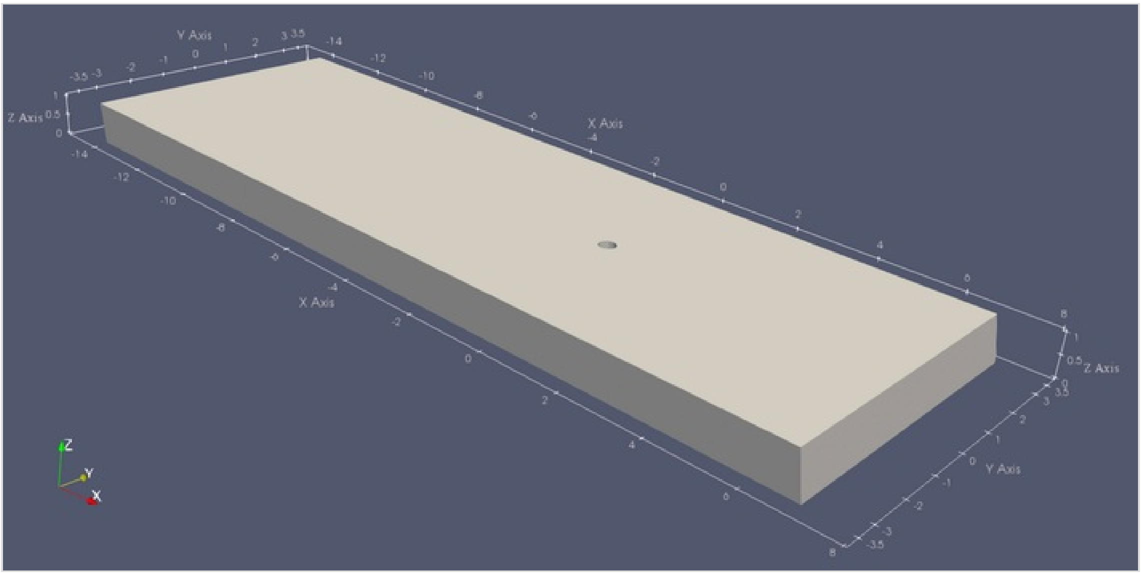

We investigated the wall shear stress ($\tau_w = \sigma \cdot \boldsymbol{n}$) around the stalagmite (which is represented as a fixed wall in the center of the domain) to estimate the effect of flowing ice on the structural integrity of the stalagmite. As shown in Table 3, the maximum wall shear stress resulting from ice deformation around the stalagmite never exceeds about 50 kPa. It is interesting to note that the highest wall shear stress occurs on the lateral downstream side of the stalagmite at the interface with the ground (Fig. 4).

Table 3 Maximum wall shear stresses due to ice flow at the stalagmite base (bottom 0.5 m).

h_{ice} (m)	β_{ice} (°)	Max. τ_w (kPa)
1.0	10	15.9
1.0	20	27.1
1.0	40	48.4
2.0	10	16.1
2.0	20	27.0
2.0	40	46.6

Figure 4

Image illustrating the magnitude of wall shear stress along the walls near the bottom of the stalagmite for $h_{ice} = 2.0$ m and $\beta_{ice} = 40°$. The stalagmite is represented as a hollow cylinder, the base of which is shown by the oval cross-section. Values for the wall shear strength are shown in Pa. Only computational cells at the stalagmite wall are displayed in color.

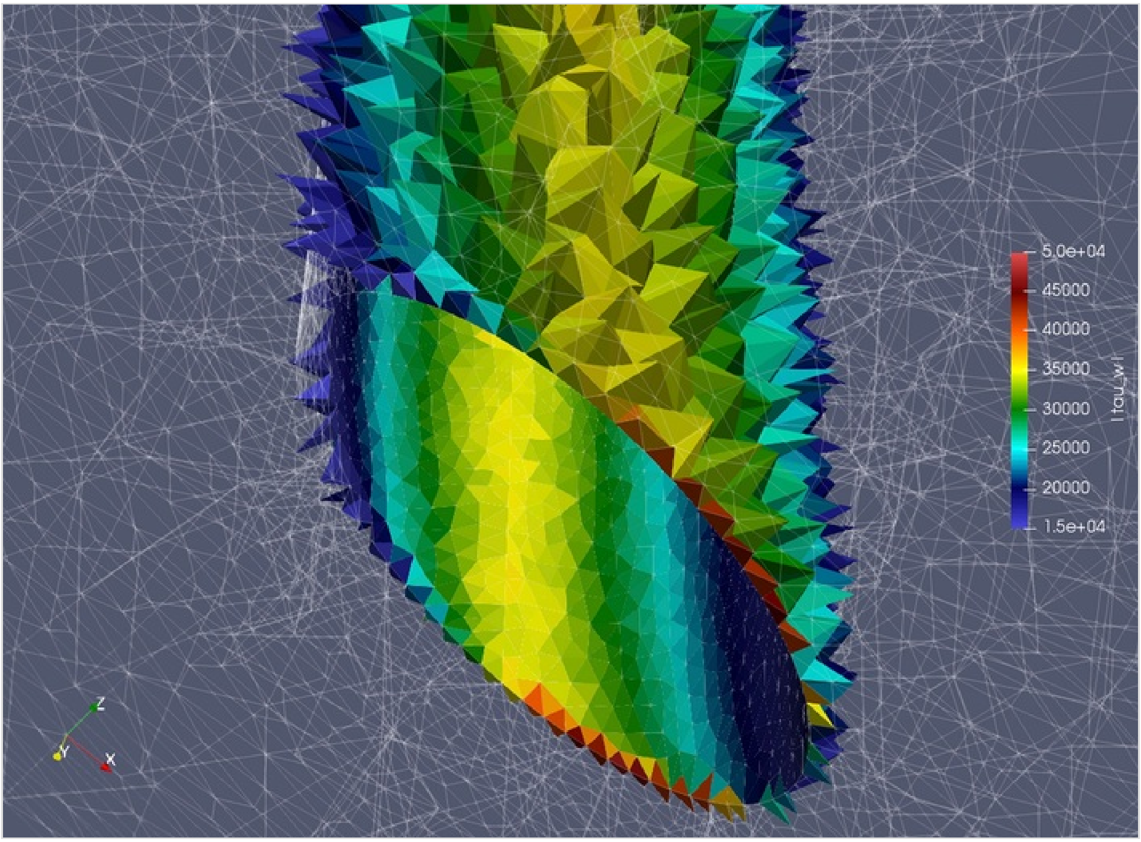

SECTION3

Thermoelasticity of a stalagmite surrounded by ice

To compare the thermoelastic stresses with the wall shear stresses caused by ice deformation, we performed a series of numerical experiments that solve Eq. (4) for different ice geometries and stalagmite dimensions and for different locations in this hypothetical cave chamber. In this model, the stalagmite (connected to the cave floor) is part of the deformation model (Eq. (4)), so we can investigate the stresses inside the stalagmite. Considering the relative temperature change term in Eq. (4) (T–T_0), its linear contribution to the overall stress regime is obvious. Therefore, we simulated only T–$T_0 = 1$ K, since the computed results can be easily multiplied by any T–T_0 value to obtain the effect of a given temperature increase or decrease on the stress regime.

We varied the stalagmite diameter ($d_s = [0.1, 0.2]$ m) and the ice thickness ($h_{ice} = [0.2, 0.5]$ m) for stalagmite location and side wall configurations as described in Table 4 and shown in Fig. 5. We applied a displacement-free boundary condition to both the side walls and the bottom, simulating the cave walls. The ice surface on the top can freely move. Removal of the right side wall (scenarios b through e) allows free deformation of the ice body in the positive X direction. Shortening the distance between the stalagmite and the right side of the ice body simulates scenarios where the stalagmite is closer to the edge of the ice body without changing the influence of the other three confining walls.

Table 4 Description of the five different stalagmite locations and side wall configurations tested.

Scenario	Side wall configuration	Description
a	Confined by all four side walls	Stalagmite surrounded by 1.5 m wide ice
b	Right side wall removed	Stalagmite surrounded by 1.5 m wide ice
c	Right side wall removed	Stalagmite 1.0 m away from right side wall
d	Right side wall removed	Stalagmite 0.5 m away from right side wall
e	Right side wall removed	Stalagmite 0.25 m away from right side wall

10.1038/s41598-023-34499-9

Figure 5 this Figure is almost too small in the pdf version. Make sure it is big enough so that the numbers can be read well.

Vertical displacement for two investigated scenarios of 1 K warming: the ice body is confined by all four side walls (left), and the right side wall is removed (right). The z axis is exaggerated by 10,000× to visualize the ice expansion. The stalagmite is located in the center of the ice block. $d_s = 0.1$ m, $h_{ice} = 0.2$ m. Note vertical ice expansion around the stalagmite.

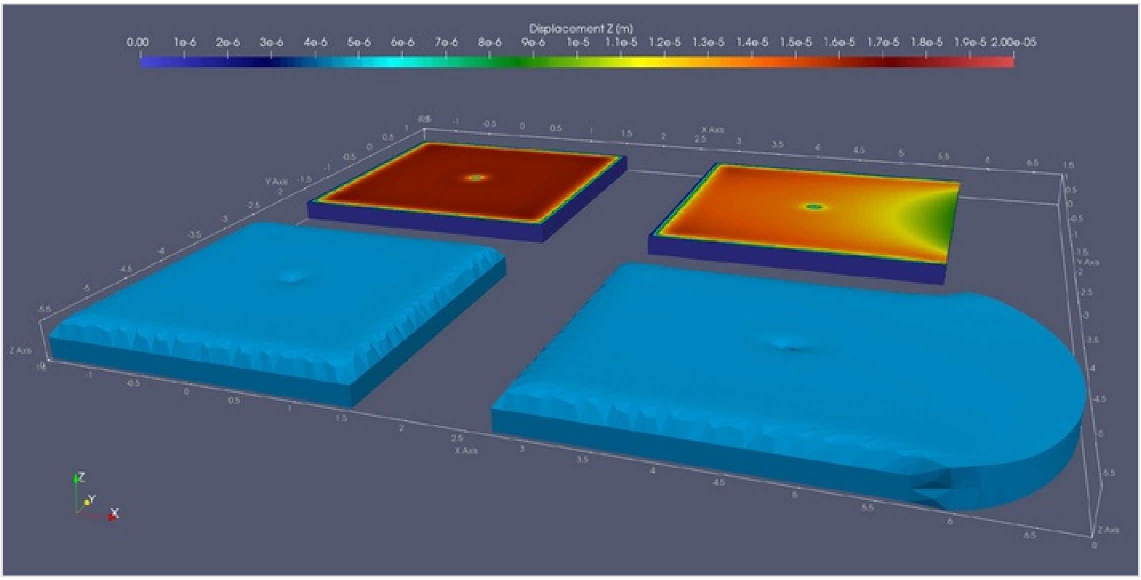

The highest stresses at the stalagmite base occur in the principal stress components, especially in the z-direction (σ_{zz}), as shown in Fig. 6. We examined these stresses at two locations at the stalagmite base, both on the side facing the removed wall in scenarios b-e (labeled “us” in Fig. 7) and on the opposite side (labeled “ds” in Fig. 7). Even a small warming of the ice body by 2 K leads to stresses sufficient to reach the tensile strength of stalagmite calcite. Basal stress has a nearly linear relationship with the diameter of the stalagmite and the thickness of the surrounding ice, i.e. if the ice thickness doubles or the diameter of the stalagmite halves, the stress is twice as high.

Figure 6

Cross section through the stalagmite encased in the ice, looking at principal stresses (in Pa) along three directions (σ_{xx}, σ_{yy} and σ_{zz}) for $d_s = 0.1$ m and $h_{ice} = 0.2$ m, a warming by 1 K and scenario a (see Table 4 [2 should read Table]). Note highest stress at the base of the stalagmite (up to 2.2 MPa).

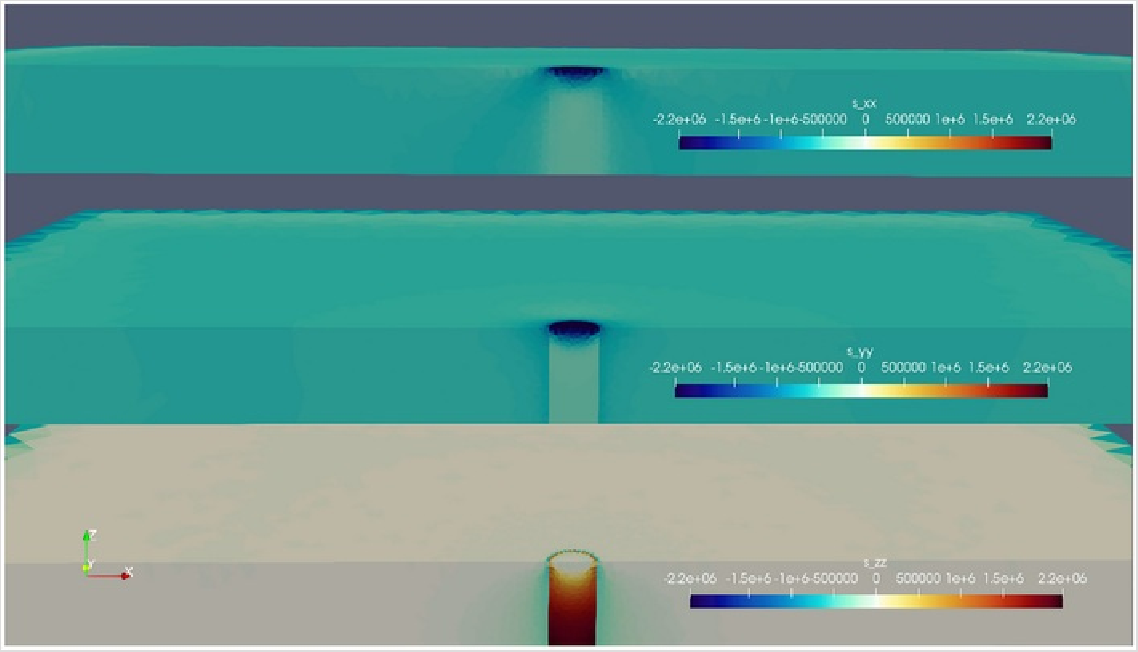

Figure 7 Compared to the previous Figs. this Fig. is pretty large in the pdf version; could be made smaller

Maximal vertical stresses at the stalagmite base for different stalagmite locations, wall scenarios, two different stalagmite diameters (d_s), two different ice thicknesses (h_{ice}) and three warming scenarios of 1, 2 and 3 K. "us" denotes "upstream side" and "ds" denotes "downstream side". Results of scenarios a to e (see Table 4 [2 should read Table]) are displayed on the x axes. The horizontal black line and the grey bar mark the mean of the tensile stress determined for stalagmite calcite from Obir cave including the standard deviation, respectively (see Fig. 2).

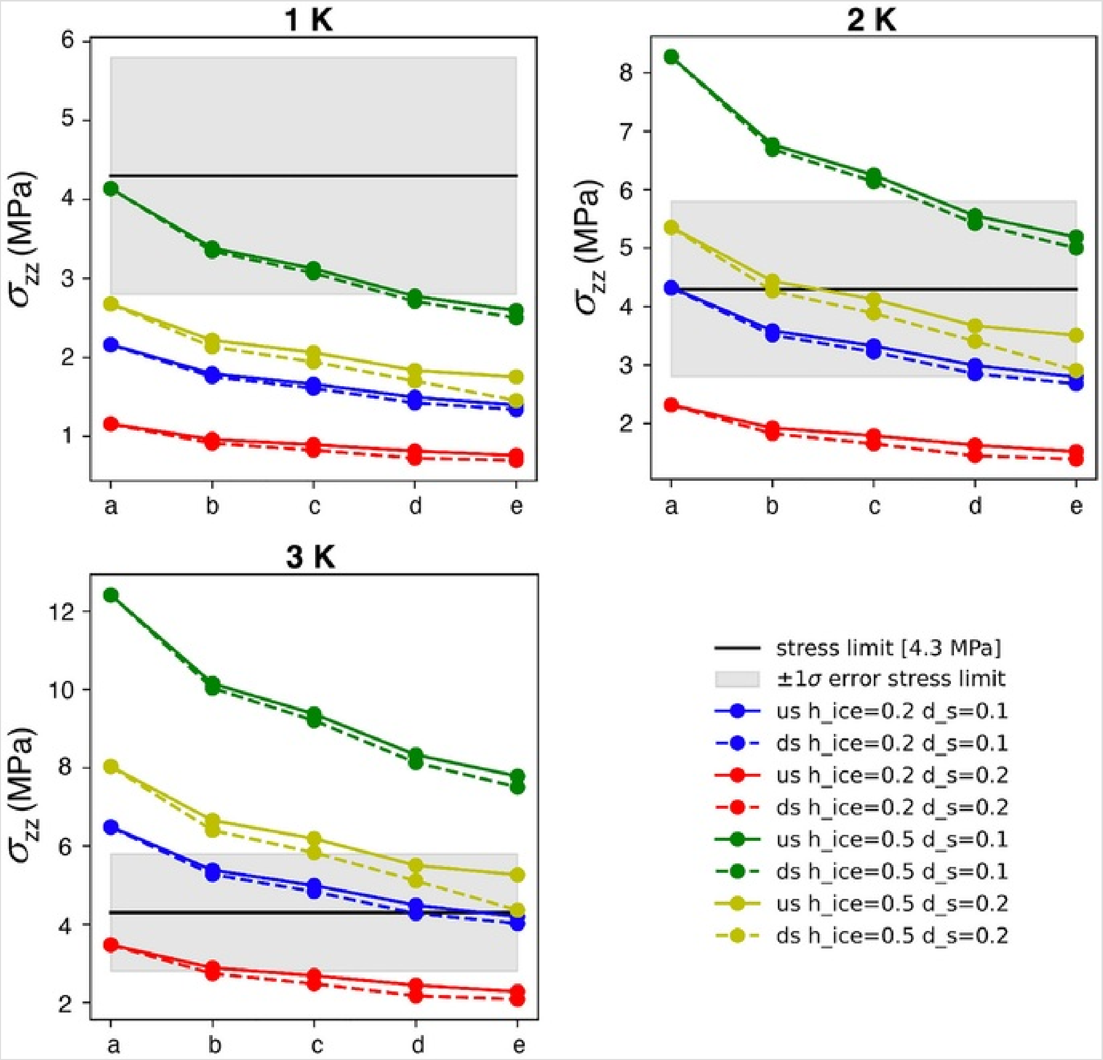

SECTION3

Occurrence and age of fractured stalagmites

The observations made in the Banane system show that in-situ stalagmites were subjected to stresses that exceeded their strength, causing one or even more cracks to form, sometimes associated with a small lateral displacement. The number of identified fractured stalagmites is a minimum estimate because stalagmites that may have fallen and are no longer in place were not considered, and some fractures of in-situ stalagmites may be obscured by younger calcite. No Holocene stalagmite exhibited this type of mechanical damage. Although the exact timing of the fracture and partial shearing cannot be determined directly by ^{230}Th dating, it can be reliably constrained by dating samples that bracket this event: pre-fracture calcite represents a maximum age, while the first post-fracture calcite layer provides a minimum age for the event. The ages of the post-fracture calcite from Banane cave are consistent with deformation prior to about 12.5 ka, i.e. before the Younger Dryas. Pre-fracture samples provide less stringent constraints and indicate that the deformation is younger than the Last Interglacial (3 samples) and younger than 44 ka (one sample – Fig. 1). These ages for pre-fracture calcite are consistent with ages for stalagmites in other parts of Obir Caves, indicating preferential growth during interglacials and no growth during glacial maxima[23, 25] (and unpublished data by the authors). The determined age range of 12.5 < X < 44 ka is also consistent with data from CCC collected in the same parts of the cave, demonstrating the presence of cave ice from at least 26.6 ± 0.2 to 23.5 ± 0.1 ka BP[24]. The spatial association of broken speleothems and CCC, as well as their age distribution, suggest damage during the Last Glacial Maximum.

10.1038/s41598-023-34499-9

That these deformation features are due to ice rather than earthquakes is consistent with the absence of neotectonic features such as fault planes in those parts of the Banane cave where the fractured stalagmites occur. Furthermore, experimental and numerical studies have shown that extremely high ground accelerations would be required to fracture such rather short (< 1 m high) and thick stalagmites (in contrast to high and thin candlestick-type stalagmites[5, 27]).

A possibly unique aspect of the Obir Caves is that there is also evidence of brittle deformation along major fault planes affecting speleothems in other parts of this large system. In Wartburghalle and Lange Grotte, a large hall and a major passage, respectively, located about 170–260 m SE of the Banane system, two fault planes showing slickensides and Riedel shear indicators can be traced for tens of meters[8]. Evidence of damage to speleothems from sliding along these faults includes a stalactite that has fallen, overturned, and embedded in a flowstone, fallen and rotated pieces of drapery, two large broken stalagmites, and a 2 m-high dripstone column that has some damage in its central portion. Deformation of the latter formation was attributed to the 1976 Friuli earthquake with a magnitude of 6.7 and a hypocentral distance of about 100 km. The other speleothem damage was dated to between 41.8 ± 1.3 and 5.7 ± 1.2 ka[8]. However, none of the feature types reported from Wartburghalle and Lange Grotte are comparable to those observed in the Banane system. Only two broken stalagmites in Lange Grotte are approximately similar. One stalagmite there is 2.1 m high and 15 cm wide; its upper part broke off and remained titled. This event has been dated to between 6.3 ± 0.2 and 5.7 ± 1.2 ka[8] and is thus much younger than the fracturing observed in the Banane system. The second stalagmite, 1.2 m high and 35 m thick, was broken into three pieces but undated, and the cause of its breakage seems unclear.

That the fractured stalagmites in the Banana system are due to ice rather than seismic shaking is supported not only by the presence of CCC, confirmed by the dating of calcite formed before and after deformation of the stalagmites, but also by the conspicuous occurrence of these damaged stalagmites themselves: they are all located on steep slopes and/or near cave walls. This suggests a fundamental role of the former cave ice and its dynamics in the destruction of these formations.

SECTION3

Stalagmite deformation by ice

Our modelling results show that the previously held view that stalagmites are broken and/or sheared by the flow of cave ice bodies[13, 14] is incorrect: the stress regime generated by internal ice deformation does not have the potential to shear stalagmites. Even in the extreme scenario of the highly stressed end member of $h_{ice} = 2$ m and $\beta_{ice} = 40°$ with a fully sliding ice base, the maximum wall shear stresses do not exceed 0.05 MPa. This is two orders of magnitude lower than the experimentally measured value of 4.3 ± 1.5 MPa at which failure must occur (Table 2 3 should read Table). The results show a clear dependence of the stress on the inclination of the ice body (Table 3), while doubling of the ice thickness has no significant effect on the resulting wall stresses. The observed slight decrease in wall stress with increasing ice thickness at the base of the stalagmite is most likely due to a slight shift in the stress regime towards the upper region of the stalagmite. Since we were interested in the base stresses where shearing failure is observed in nature, we did not investigate this stress change further.

In contrast to internal ice deformation caused by ice flow on a sloping substrate, thermoelastic stresses within an ice cave body have a high potential to cause stalagmite failure. The stress distribution in Fig. 6 shows that the differences in thermal expansion coefficients cause a sharp vertical jump in stress between the stalagmite and the surrounding ice. This results in vertical shear on the stalagmite at its base or possibly higher up where the surrounding ice lifts the stalagmite as it expands with increasing temperature. Figure 7 shows these stresses for five scenarios and a relative temperature increase of 1, 2 and 3 K. Scenario a yielded the highest stresses because the ice body is completely enclosed by four surrounding walls. Therefore, the ice can only expand upward. This deformation is greatest in the domain center where the stalagmite is located. In scenarios b to e, the maximum stress decreases somewhat because the confinement of the fourth wall is missing.

Depending on the relative temperature increase, several of our scenarios would result in a vertical shear failure near the base of the stalagmite and a subsequent vertical displacement of the stalagmite. In scenario e for $h_{ice} = 0.5$ m and $d_s = 0.2$ m, we observe a significant difference between the stress magnitude on the "upstream" and "downstream" sides of the stalagmite. This relative stress difference would cause rotational motion and thus a slight rotational shear of the stalagmite near its base.

Our modelling results show that stalagmites are not sheared from their base by lateral ice flow or lateral thermoelastic expansion and contraction of the ice; instead they are lifted by warming and expansion of the surrounding ice (and vertically compressed as the ice cools and contracts). We suspect that multiple cooling and warming cycles below 0 °C weaken the stalagmites, eventually causing them to break. As a result, the stalagmites are locally slightly displaced laterally by ice flow, sometimes falling to the ground, or are cemented in an upright position by calcite.

Fractured stalagmites that are still in situ are not only a diagnostic indicator of the past presence of cave ice, but also indirectly

witness significant paleotemperature fluctuations below the freezing point in karst rocks resulting in fracturing of even large and thick stalagmites.

SECTION3

Link to glacial climate variability

The simultaneous occurrence of damaged stalagmites and CCC in now ice-free caves reflects a specific temperature history: (1) cooling of the cave below 0 °C, (2) accumulation of floor ice by freezing of water entering the cave, (3) warming of the cave and its ice deposit by up to a few degrees, but still below 0 °C, leading to breakage of stalagmites encased in the ice, locally accompanied by small a lateral displacement and/or rotation, (4) further slow warming under fairly stable thermal conditions just below 0 °C, infiltration of drip water and the local precipitation of CCC in pools of water in the floor ice, (5) major warming beyond 0 °C, deglaciation of the cave and overturning of some fractured stalagmites. Critical to effective fracturing of stalagmites are (a) severe initial cooling (and subsequent warming in the subzero temperature range causing ice expansion), (b) sufficiently thick floor ice (which scales linearly with thermoelastic stresses acting on the stalagmite), and (c) multiple cooling and warming cycles at subzero temperatures that weaken even large stalagmites. **AQ1**

This temperature evolution of the shallow subsurface argues for a paleoclimate characterized not only by strong cooling to temperatures below 0 °C (in the case of Obir caves, by more than 6 °C compared to the present-day cave temperature), but also by temperature fluctuations that resulted in cycles of ice contraction and expansion of cave ice. This is consistent with the magnitude and pattern of climate change during glacial periods and especially during glacial maxima. These periods were characterized by severe cooling (on the order of 10 °C in the Alps[28, 29]) and by a high degree or climate variability, including Heinrich stadials and minor events of iceberg discharge into the North Atlantic, which influenced the climate in Europe[30, 31, 32]. Permafrost was widespread in the Central Europe between the southern margin of the Scandinavian ice sheet and the Alpine ice sheet, including the southern part of England[20]. It is therefore not surprising that most reports of presumably ice-damaged speleothems are from caves extending from Slovakia[16] and the Czech Republic[17] to Germany[14, 33, 34, 35], Belgium[36] and southern England[15]. No systematic dating of damaged stalagmites at these sites has been performed, but the ^{230}Th ages of the associated CCC range mostly from about 40 to 15 ka[37, 38, 39, 40], demonstrating the presence of ice in these (currently ice-free) caves. **AQ2**

SECTION1

Conclusions

The Obir Caves provide evidence of mechanical fracturing of stalagmites that are spatially associated with CCC deposits. This damage is younger than about 44 ka and older than 12.5 ka, which covers the age range of CCC from the first half of the Last Glacial Maximum.

Numerical modelling indicates that ice deformation under gravity cannot cause stalagmite breakup in ice caves because the resulting stresses are two orders of magnitude less than required. In contrast, ice expansion (and contraction) due to changes in the thermal regime has the potential to break off stalagmites. Depending on the ice thickness and the diameter of the stalagmite, a temperature increase of as little as 2 K can cause vertical shear stresses strong enough to vertically shear off a stalagmite near its base. If the cave ice body is not confined by surrounding walls, i.e. the cave chamber is only partially filled with ice, vertical shear stress gradients can form at the stalagmite base, resulting in effective rotational stresses.

Vertical uplift of stalagmites by expanding ice – rather than breakage by lateral ice flow as previously assumed – provides a physical explanation for the wide-spread damage to speleothems in mid-latitude caves during cold climate periods of the Pleistocene. The observed characteristics of ice-damaged stalagmites, which exhibit fractures, some of which are associated with minor lateral displacement but are still in-situ, i.e. in growth position, differ from the damage caused by strong earthquakes on speleothems growing on active fault zones in other parts of this cave system. In this respect, the Obir Caves represent a rare example where both types of speleothem deformation can be studied and chronologically constrained by ^{230}Th dating.

SECTION1

Methods

SECTION2

Field work

We examined fractured stalagmites in the same chambers of the Banane system where samples of CCC from the Last Glacial

Maximum were recovered[24]. We documented the deformation features and drilled short cores (7 mm diameter) to sample the pre- and post-deformation calcite for ^{230}Th dating.

We also extracted a few larger broken stalagmites for laboratory tests of the mechanical properties of speleothem calcite. Samples for these tests were cut from the interior of these stalagmites as prismatic blocks with edge lengths of 20 to 40 mm and heights from 50 to 75 mm.

SECTION2

Age determination

The drill cores were examined macroscopically and samples for dating were obtained from these cores using a hand-held dental drill, operated in a laminar-flow hood. The samples were prepared and ^{230}Th dated at the Isotope Laboratory of Xi'an Jiaotong University, using standard chemistry procedures[41] to separate U and Th. U and Th isotopes were measured using a multi-collector inductively coupled plasma mass spectrometer (ThermoFinnigan Neptune Plus) equipped with a MasCom multiplier behind the retarding potential quadrupole in peak-jumping mode. Instrumentation and standardization are described in Cheng et al.[2]. ^{230}Th ages were calculated using decay constants of Jaffey et al.[42] and Cheng et al.[2].

SECTION2

Laboratory measurements of mechanical properties of stalagmites

Measurements of mechanical properties, i.e. tensile and compressive strength – the Young's modulus and Poisson ratio in tension and compression, respectively – were performed on prismatic samples were cut from large broken stalagmites. For the measurements in tension aluminum posts with a thread in the center were glued to the face sides (using X60 glue from HBK company) and mounted in the testing machine. For the compression measurements, the samples were placed between two steel plates in the testing machine (AGX-plus from Shimadzu company, maximum load 50 kN).

To measure the Young's modulus and Poisson ratio, specimens were subjected to three loading cycles. The loading rate was adjusted so that both the maximum and minimum loading of each cycle were achieved within 12 to 15 s. After loading and unloading, the corresponding load was held constant for 30 s. Data were recorded using a Spider8 data acquisition system (HBK company) at a frequency of 5 Hz.

The change in length was measured with two DD1 displacement transducers (HBK company) attached directly to the specimen. In addition, a T-Rosette (1-XY11-6/120, HBK company) was glued to the specimen at half height to determine the linear and lateral strain. The Young's modulus was determined from the recorded data of three loading cycles. This parameter was calculated for each loading and unloading step and an average value was determined for the specimen. The Poisson ratio was calculated from the average values of the longitudinal and lateral strain of each loading and unloading period and a mean value was determined.

SECTION2

Numerical modelling

We investigated two possible mechanisms associated with ice formation in a cave: (a) ice deformation under gravity, and (b) expansion/contraction of ice due to temperature changes. We used finite-element models for thermoelasticity and full-Stokes ice flow to determine the relative contributions of these two processes to basal stresses around stalagmites.

Two finite element models (detailed below), implemented in the FEniCS (https://fenicsproject.org[43, 44]) framework, were used. This framework allows for an easy definition of the model physics equations in variational form and subsequently derives a functional FEM model code (including boundary condition treatments), which can be used for computations. Symbols used in both models and values of standard parameters are listed in Table 5.

Table 5 Notations and parameters used in the numerical models.

Symbol	Description	SI units	Value
A	Glen flow law parameter for temperate ice	$Pa^{-3}\ s^{-1}$	2.40×10^{-24}
C bold + italics	Stiffness tensor	–	–
d_s	Stalagmite diameter	m	–
E_i	Young's modulus for ice	Pa	9.5×10^{9}
E_s	Young's modulus for stalagmite	Pa	7.62×10^{10}
g	Gravity magnitude	$m\ s^{-2}$	9.81
h_{ice}	Uniform ice thickness in cave	m	–
n	Glen flow law non-linearity factor	–	3.00
n bold + italics	Wall normal unit vector	–	–
p	Fluid pressure	Pa	–
ΔT	$= T–T0$, relative temperature change	K	–
u bold + italics	Ice flow velocity field	$m\ s^{-1}$	–
x	Displacement	m	–
α_i	Thermal expansion coefficient for ice	K^{-1}	4.0×10^{-5}
α_s	Thermal expansion coefficient for stalagmite	K^{-1}	8.0×10^{-6}
β_{ice}	Slope angle of cave bottom	Degree	–
δ_{ij}	Kronecker delta	–	–
$\dot{\varepsilon}$	Effective strain rate for ice	S^{-1}	–
ε bold + italics	Strain tensor	–	–
η	Effective Glen viscosity	Pa s	–
λ	First Lamé parameter	–	–
μ	Second Lamé parameter	–	–
v_i	Poisson ratio for ice	–	0.33
v_s	Poisson ratio for stalagmite	–	0.27
ρ_i	Ice density	$kg\ m^{-3}$	910.00
σ bold + italics	Stress tensor	Pa	–
τ_w	Wall shear stress	Pa	–

Ice deforms under gravity like a non-linear viscous fluid, with a strain-dependent effective viscosity η that follows the relationship of Glen[45]. We considered the full stress–strain relation in our simulations and solved the corresponding Stokes equation In the equation 1 below please make the symbols "u" (2x) and "g" bold+italics :

$$-\nabla \cdot \left[\eta\left(\nabla u + \nabla u^T\right)\right] + \nabla p = \rho g. \quad (1)$$

Here In this sentence please make the symbols "u" and "g" bold+italics u is the velocity field vector, p the fluid pressure, ρ the fluid density and g the driving force, gravity, and Glen's effective viscosity η defined as

$$\eta = \frac{1}{2} A^{-1/n} \dot{\varepsilon}^{(1-n)/n}, \quad (2)$$

denotes the flow law parameter and n the non-linearity factor. The term $\dot{\varepsilon} = \sqrt{\frac{1}{2}\dot{\varepsilon}_{ij}\dot{\varepsilon}_{ji}}$ is the effective strain rate. A more detailed description of the model implementation can be found in Jarosch[46] and a software framework update to utilize FEniCS is detailed in Wirbel et al.[47].

We describe the volumetric changes and resulting stresses within the ice and the stalagmite caused by ambient temperature changes (ΔT) using a linearized thermoelastic constitutive equation In the equation 3 below please make the symbols "σ", "C" and "ε" bold+italics :

$$\sigma = C : [\varepsilon - \alpha \Delta T], \quad (3)$$

where $\boldsymbol{\sigma}$ is the stress tensor, **C** bold + italics
the stiffness tensor and $\boldsymbol{\varepsilon}$ the strain tensor. Rewriting Eq. (3) in more detail with index notation and expressing the strains in terms of displacements (x) leads to

$$\sigma_{ij} = \mu\left(x_{i,j} + x_{j,i}\right) + \left[\lambda x_{k,k} - \alpha\left(3\lambda + 2\mu\right)\left(T - T_0\right)\right]\delta_{ij}, \quad (4)$$

with λ and μ being the Lamé parameters and α the thermal expansion coefficient[48]. The relative temperature change of ice and speleothem calcite is now expressed with respect to a reference temperature (T_0) and δ_{ij} is the Kronecker delta. Both Lamé parameters can be related to the Young's modulus (E) and the Poisson ratio (ν):

$$\lambda = \frac{E\nu}{\left(1+\nu\right)\left(1-2\nu\right)}, \quad (5)$$

$$\mu = \frac{E}{2\left(1+\nu\right)}, \quad (6)$$

We implemented Eq. (4) in FEniCS, which is a novel contribution of this work, to utilize the same FE solving library as well as pre- and post-processing routines as for the ice flow calculations.

Supplementary Information

The online version contains supplementary material available at https://doi.org/10.1038/s41598-023-34499-9.

Publisher's note

Springer Nature remains neutral with regard to jurisdictional claims in published maps and institutional affiliations.

Acknowledgements

On-site support during cave work was provided by T. Racine and A. Langer. We thank two referees for their constructive reviews.

Author contributions

C.S. designed the research, wrote the manuscript, and prepared the figures. A.J. performed numerical modelling, A.S. performed mechanical stress measurements, G.K. assisted in field work and contributed to the final version of the manuscript, H.Z. performed ^{230}Th dating. All authors reviewed the manuscript.

Funding

The Austrian Science Fund (FWF grant P 318740) partially supported this research Replace this sentence by:
This research was funded in part by the Austrian Science Fund (FWF), grant P318740. For the purpose of open access, the author has applied a CC BY public copyright licence to any Author Accepted Manuscript version arising from this submission.
.

Data availability

All data generated or analysed during this study are included in this published article and its Supplementary Information file or are available from the first author upon request.

Competing interests

The authors declare no competing interests.

SECTION1

Supplementary Information

Supplementary Information.

References

1. Fairchild, I. J. & Baker, A. *Speleothem Science. From Processes to Past Environments* (Wiley-Blackwell, 2012).

2. Cheng, H. *et al.* Improvements in ^{230}Th dating, ^{230}Th and ^{234}U half-life values, and U-Th isotopic measurements by multi-collector inductively coupled plasma mass spectrometry. *Earth Planet. Sci. Lett.* **371–372**, 82–91 (2013).

3. Kagan, E. J., Agnon, A., Bar-Matthews, M. & Ayalon, A. Dating large infrequent earthquakes by damaged cave deposits. *Geology* **33**, 261–264 (2005).

4. Plan, L. *et al.* Neotectonic extrusion of the Eastern Alps: Constraints from U/Th dating of tectonically damaged speleothems. *Geology* **38**, 483–486 (2010).

5. Gribovszki, K. E. *et al.* Estimating the upper limit of prehistoric peak ground acceleration using an in situ, intact and vulnerable stalagmite from Plavecká priepast cave (Detrekői-zsomboly), Little Carpathians Slovakia - first results. *J. Seismol.* **21**, 1111–1130 (2017).

6. Pace, B. *et al.* A large paleoearthquake in the central Apennines, Italy, recorded by the collapse of a cave speleothem. *Tectonics* **39**, e2020TC006289. https://doi.org/10.1029/2020TC006289 (2020).

7. Szczygieł, J. *et al.* Quaternary faulting in the Western Carpathians: Insights into paleoseismology from cave deformations and damaged speleothems (Demanova Cave System, Low Tatra Mts). *Tectonophysics* **820**, 229111. https://doi.org/10.1016/j.tecto.2021.229111 (2021).

8. Baroň, I. *et al.* Three large prehistoric earthquakes in the Eastern Alps evidenced by cave rupture and speleothem damage. *Geomorphology* **408**, 108242. https://doi.org/10.1016/j.geomorph.2022.108242 (2022).

9. Grasemann, B., Plan, L., Baroň, I. & Scholz, D. Co-seismic deformation of the 2017 Mw 6.6 Bodrum-Kos earthquake in speleothems of Korakia Cave (Pserimos, Dodecanese, Greece). *Geomorphology* **402**, 108137. https://doi.org/10.1016/j.geomorph.2022.108137 (2022).

10. Brady, J. E., Scott, A., Neff, H. & Glascock, M. D. Speleothem breakage, movement, removal, and caching: An aspect of ancient Maya cave modification. *Geoarchaeology* **12**, 725–750 (1997).

11. Jaubert, J. *et al.* Early Neanderthal constructions deep in Bruniquel Cave in southwestern France. *Nature* **534**, 111–114 (2016).

12. Kempe, S. Natural speleothem damage in Postojnska Jama (Slovenia), caused by glacial cave ice? A first assessment. *Acta Carsol.* **33**, 265–289 (2004).

13. Gilli, E. Glacial causes of damage and difficulties to use speleothems as palaeoseismic indicators. *Geodin. Acta* **17**, 229–240 (2004).

14. Kempe, S., Bauer, I. & Dirks, H. Glacial cave ice as the cause of wide-spread destruction of interglacial and interstadial speleothem generations in central Europe. *Proc. 15th Intern. Congr. Speleol.* **2**, 1026–1031 (2009).

15. Lundberg, J. & McFarlane, D. A. Cryogenic fracturing of calcite flowstone in caves: theoretical considerations and field observations in Kents Cavern, Devon, UK. *Int. J. Speleol.* **41**, 307–316 (2012).

16. Orvošová, M., Vlèek, L., Holúbek, P. & Orvoš, P. Frost and cave ice action as a cause of speleothem destruction during glacial: Examples from selected caves in Slovakia. *Slovenský Kras Acta Carsol. Slovaca* **50**, 157–172 (2012).

17. Žák, K. *et al.* Cryogenic carbonates and cryogenic speleothem damage in the Za Hájovnou Cave (Javoříčko Karst, Czech Republic. *Geol. Q.* **62**, 829–839 (2018).

18. Žák, K. *et al.* Cryogenic mineral formation in caves. In *Ice Caves* (eds Perşoiu, A. & Lauritzen, S.-E.) 123–162 (Elsevier, 2018).

19. Nigst, P. R. *et al.* Early modern human settlement of Europe north of the Alps occurred 43,500 years ago in a cold steppe-type environment. *PNAS* **111**, 14394–14399 (2014).

20. Vandenberghe, J. *et al.* The Last Permafrost Maximum (LPM) map of the Northern Hemisphere: permafrost extent and mean annual air temperatures, 25–17 ka BP. *Boreas* **43**, 652–666 (2014).

21. Moine, O. *et al.* The impact of Last Glacial climate variability in west-European loess revealed by radiocarbon dating of fossil earthworm granules. *PNAS* **114**, 6209–6214 (2017).

22. Spötl, C. *et al.* Stable isotope imprint of hypogene speleogenesis: Lessons from Austrian caves. *Chem. Geol.* **572**, 120209. https://doi.org/10.1016/j.chemgeo.2021.120209 (2021).

23. Spötl, C. *et al.* Tropfstein-Forschung in österreichischen Höhlen—Ein Überblick. *Jahrb. Geol. Bundesanst.* **147**, 117–167 (2007).

24. Spötl, C., Koltai, G., Jarosch, A. & Cheng, H. Increased autumn and winter precipitation during the Last Glacial Maximum in the European Alps. *Nat. Commun.* **12**, 1839. https://doi.org/10.1038/s41467-021-22090-7 (2021).

25. Fairchild, I.J. *et al.* Petrology and geochemistry of annually laminated stalagmites from an Alpine cave (Obir, Austria): seasonal cave physiology. In Pedley, H.M. & Rogerson, M. (eds) *Tufas and Speleothems: Unravelling the Microbial and Physical Controls*, *Geol. Soc. London Spec. Publ.* **336**, 295–321 (2010).

26. Honiat, C. *et al.* A paleoprecipitation and paleotemperature reconstruction of the Last Interglacial in the southeastern Alps. Clim. Past Discuss. https://doi.org/10.5194/cp-2022-78 (2022).

27. Mendecki, M. & Szczygieł, J. Physical constraints on speleothem deformations caused by earthquakes, seen from a new perspective: Implications for paleoseismology. *J. Struct. Geol.* **126**, 146–155 (2019).

28. Haeberli, W., Rellstab, W. & Harrison, W. D. Geothermal effects of 18 ka ice conditions in the Swiss Plateau. *Ann. Glaciol.* **5**, 56–60 (1984).

29. Kuhlemann, J., Rohling, E. J., Kubik, P., Ivy-Ochs, S. & Kucera, M. Regional synthesis of Mediterranean atmospheric circulation during the Last Glacial Maximum. *Science* **321**, 1338–1340 (2008).

30. Moseley, G. E. *et al.* NALPS19: Sub-orbital-scale climate variability recorded in northern Alpine speleothems during the last glacial period. *Clim. Past* **16**, 29–50 (2020).

31. Sirocko, F. *et al.* Muted multidecadal climate variability in central Europe during cold stadial periods. *Nat. Geosci.* **14**, 651–658 (2021).

32. Prudhomme, C. *et al.* Millennial-timescale quantitative estimates of climate dynamics in central Europe from earthworm calcite granules in loess deposits. *Commun. Earth Environ.* https://doi.org/10.1038/s43247-022-00595-3 (2022).

33. Kempe, S., Fenzlein, R. & Schapbach, H. The Geisloch, a well preserved hypogene cave: Statistical analysis of pronounced glacial speleothem damage and first U/Th dating of cave bear remains to older than 80 ka BP (Oberfellendorf, Franconian Alb, Germany). *e-Res. Rep. Museum Burg Golling* **4**, 1–10 (2022).

34. Richter, D. K. *et al.* Zerbrochene Höhlensinter und Kryocalcite als Indikatoren für eiszeitlichen Permafrost im Herbstlabyrinth-Adventhöhle-System bei Breitscheid-Erdbach (N-Hessen). *Höhle* **62**, 31–45 (2011).

35. Schapbach, H. Natürliche Sinterzerstörung in fränkischen Höhlen - auf der Spur des verschwundenen Eises. *Fränkische Höhlenspiegel* **65**, 22–35 (2023).

36. Cadorin, J. F. *et al.* Modelling of speleothem failure in the Hotton cave (Belgium). Is the failure earthquake induced?. *Netherl. J. Geosci. Geol. Mijnbouw* **80**, 315–321 (2001).

37. Žák, K. *et al.* Coarsely crystalline cryogenic cave carbonate—A new archive to estimate the Last Glacial minimum permafrost depth in Central Europe. *Clim. Past* **8**, 1821–1837 (2012).

38. Orvošová, M., Deininger, M. & Milovsky, R. Permafrost occurrence during the Last Permafrost Maximum in the Western Carpathian Mountains of Slovakia as inferred from cryogenic cave carbonate. *Boreas* **43**, 750–758 (2014).

39. Richter, D. K., Meyer, S., Scholz, D. & Immenhauser, A. Multiphase formation of Weichselian cryogenic calcites, Riesenberg Cave (Süntel/NW Germany). *Z. Dt. Ges. Geowiss.* **164**, 353–367 (2013).

40. Richter, D. K. *et al.* Weichselzeitliche Kryocalcite als Hinweis für Eisseen in der Hüttenbläserschachthöhle (Iserlohn/NRW). *E&G Quat. Sci. J.* **64**, 67–81 (2015).

41. Edwards, R. L., Chen, J. H. & Wasserburg, G. J. ^{238}U–^{234}U-^{230}Th-^{232}Th systematics and the precise measurement of time over the past 500,000 years. *Earth Planet. Sci. Lett.* **81**, 175–192 (1987).

42. Jaffey, A. H., Flyn, K. F., Glendenin, L. E., Bentley, W. C. & Essling, A. M. Precision measurement of half-lives and specific activities of ^{235}U and ^{238}U. *Phys. Rev. C* **4**, 1889–1906 (1971).

43. Alnæs, M. S. *et al.* The FEniCS project version 1.5. *Arch. Numer. Softw.* https://doi.org/10.11588/ans.2015.100.20553 (2015).

44. Logg, A., Mardal, K.-A. & Wells, G. Automated solution of differential equations by the Finite Element Method. In *Lecture Notes in Computational Science and Engineering* Vol. 84 (eds Logg, A. *et al.*) (Springer, 2012).

45. Glen, J. W. The creep of polycrystalline ice. *Proc. R. Soc. A* **228**, 519–538 (1955).

46. Jarosch, A. H. Icetools: A full Stokes finite element model for glaciers. *Comput. Geosci.* **34**, 1005–1014. https://doi.org/10.1016/j.cageo.2007.06.012 (2008).

47. Wirbel, A., Jarosch, A. H. & Nicholson, L. Modelling debris transport within glaciers by advection in a full-Stokes ice flow model. *Cryosphere* **12**, 189–204. https://doi.org/10.5194/tc-12-189-2018 (2018).

48. Hetnarski, R. B. & Eslami, M. R. *Thermal Stresses* (Springer, 2008).

49. Gribovski, K. et al. Comprehensive investigation of intact, vulnerable stalagmites to estimate an upper limit on prehistoric horizontal ground acceleration. In Adam, C. et al. (ed.) *Proceedings of the Vienna Congress on Recent Advances in Earthquake Engineering and Structural Dynamics 2013*, 445, 1-10 (2013).

50. Waltham, T. Strength and stability of calcite stalactites. *Cave Karst Sci.* **39**, 37 (2012).

51. Lacave, C., Koller, M. G. & Egozcue, J. J. What can be concluded about seismic history from broken and unbroke speleothems?. *J. Earthq. Eng.* **8**, 431–455 (2004).

52. Paronuzzi, P. & Serafini, W. Stress state analysis of a collapsed overhanging rock slab: A case study. *Eng. Geol.* **108**, 65–75 (2009).

Typesetting math: 100%
